# SMP30: a promising cancer biomarker with therapeutic potential

**DOI:** 10.1515/med-2026-1447

**Published:** 2026-07-09

**Authors:** Pei Chen, Yu-ling Zhang, Ying Guo

**Affiliations:** Department of Basic Medicine, Jiangsu College of Nursing, Huai’an, China; Department of Clinical Laboratory, Huai’an Maternal and Child Health Care Hospital, Huai’an, China

**Keywords:** cellular senescence, SMP30, SASP, Ca^2+^ homeostasis, therapeutic target

## Abstract

**Introduction:**

SMP30 is a calcium binding protein closely related to cellular senescence. Low expression of SMP30 is significantly associated with malignant progression and poor prognosis in tumors, suggesting it values as a potential diagnostic biomarker and therapeutic target.

**Content:**

We systematically review recent studies on SMP30 in human tumors, highlight its role in modulating tumor cells proliferation and apoptosis, and detail mechanism of its involvement in glycolipid metabolic reprogramming as well as its impact on the tumor microenvironment and metastasis.

**Summary:**

SMP30 is involved in regulating cell cycle, maintaining intracellular homeostasis, and protecting cells from external damage. It supports cellular homeostasis and resistance to apoptosis by modulating intracellular Ca^2+^ concentrations. The reduced expression of SMP30 elevates lipid peroxidation, accelerating cellular senescence and tissue damage. Furthermore, SMP30 affects macrophage polarization and modulates the tumor microenvironment by altering their cytokine secretion profiles.

**Outlook:**

SMP30 is a key regulator of cellular senescence with significant implication in tumorigenesis. Low expression of SMP30 relates to multiple oncogenic mechanisms, including cell cycle, apoptosis, metabolic reprogramming, epithelial mesenchymal transition (EMT), and immune evasion, which shows promising potential for early diagnosis, prognostic evaluation, and targeted therapy of cancers.

## Introduction

In 1965, Hayflick proposed cellular senescence, describing how normal human fibroblasts lost their diploid cell division potential during continuous proliferation *in vitro*. After a vigorous reproductive period, cells cease mitosis and eventually die. Cellular senescence is a stable and irreversible state of cell cycle arrest, accompanied by changes in macromolecules [1]. It is also characterized by a senescence associated secretory phenotype (SASP). Factors such as telomere shortening, DNA damage, oxidative stress, and oncogene activation act as triggers which induce cellular senescence [2]. Fujita T [3] found the expression level of a protein decreased significantly with age, reaching an extremely low level in elderly (30-month-old) rats. The protein accounted for about 2 % of soluble liver protein in rats when detecting the relationship between soluble liver protein and age changes, and its expression level decreased by nearly 60–70 % in aged rats. Because of its initial estimated molecular weight of 30 kDa, it was named Senescence Marker Protein-30 (SMP30). Later precise measurements showed that its molecular weight was actually 34 kDa [4]. Cellular Ca^2+^ plays a critical role as a second messenger in hormonal signaling pathways. SMP30, a calcium-binding protein also known as regucalcin, plays a significant role in cell signaling and disease by modulating Ca^2+^ homeostasis. This protein is downregulated during aging, and one of its core biological functions is maintaining intracellular Ca^2+^ homeostasis. The intracellular Ca^2+^ concentration is closely related to various biological processes such as cell proliferation, apoptosis, migration, death and immune response. All changes in these biological behaviors are fundamental processes in normal cell carcinogenesis. The loss of Ca^2+^ homeostasis is considered as an important driving force for the occurrence and progression of malignant diseases such as tumors [5], 6], and Ca^2+^ signaling has become a highly attractive target for the development of novel anticancer drugs [7], 8]. SMP30 is closely related to many tumors, and its expression plays an important role in the initiation, progression, and treatment of tumors. Maintaining Ca^2+^ homeostasis, anti-oxidation and anti-apoptosis are the main mechanisms by which SMP30 exerts its anti-cancer function. SMP30 deficiency indirectly promotes tumor progression by affecting cell adhesion and coagulation pathways [9]. It has been found that SMP30 not only participates in the regulation of cellular senescence but also closely associated with the occurrence and progression of various tumors [10]. SMP30 plays a significant role in inhibiting cancers and could serve as a biomarker or therapeutic target for tumors, offering innovative insights for the early detection and treatment of various tumors. In this review, we present the latest developments concerning SMP30 to elucidate its function and mechanism in human tumors.

## Cellular senescence and tumors

Cellular senescence is a stable and irreversible state of cell cycle arrest, which is an important defense mechanism for the body to combat tumor progression [11]. The core anti-tumor mechanism of cellular senescence is to prevent the unlimited proliferation of potential precancerous cells or already cancerous cells. During the aging process of organisms, senescent cells secrete large amounts of inflammatory factors, cytokines, and matrix-degrading enzymes. These secretions collectively form SASP. The formation of SASP is a double-edged sword. It helps to clear senescent cells, repairs tissues, and inhibits tumors in the short term, however long-term accumulation of SASP can lead to chronic inflammation, tissue damage, and age-related diseases [12]. Therefore, regulating SASP is crucial for interventions against senescent and related pathologies. Activation of oncogenes triggers oncogene-induced senescence (OIS), a program which drives SASP formation. OIS induces stable growth arrest in cells, forming a natural barrier to tumorigenesis. This growth arrest process is enhanced by cyclin-dependent kinase inhibitors such as p16ˆINK4a and p21. Therefore, OIS is an inherent tumor suppression in cells [13]. Senescent cells undergo metabolic changes and exhibit SASP. Both the metabolic changes and SASP mediate tumor suppression in a non-cell-autonomous manner. SASP factors include chemokines, cytokines, growth factors, and enzymes, which mediate paracrine senescence induction or stable proliferation arrest in adjacent cancer cells. Some SASP factors also enhance immune surveillance by activating immune cells such as natural killer (NK) cells and macrophages, promoting clearance of senescent cells. In addition, SASP factors can induce secondary cell death of tumor cells [14]. Cellular senescence prevents cell proliferation in response to damage or stimulation, which reduces the risk of tumor transformation. Cellular senescence is considered a barrier to tumor development. However, inflammation, which promotes tumor progression, seems to contradict with this protective role. Senescent cells secrete inflammation factors which damage tissue structure or stimulate the proliferation of nearby cells. As a result, their presence creates a tissue environment to promote tumors. This environment, combined with carcinogenic mutations, increases tumor incidence with age [15]. It has been found that whether low level inflammation inhibits or promotes tumors related to secondary inflammation in stressed epithelium mainly depends on the activity of p53 [16]. p53 can sense stress signals, such as DNA damage and activate downstream target genes, inducing cellular senescence. Studies have shown that p53 suppresses cytoplasmic chromatin fragments (CCF) accumulation and its downstream inflammatory phenotype. Activation of p53 suppresses CCF formation, which links to enhanced DNA repair and genome integrity. Activated p53 in aged mice by pharmacological inhibition of MDM2 reverses transcriptomic signatures of aging and age-associated accumulation of monocytes and macrophages in liver [17]. Multiple cytokines induce senescence-related inflammatory responses (SIR) in tumors, leading to cell growth arrest [18], 19]. However, in the absence of p53, cells lose growth control and exhibit accelerated growth and invasion. It has been confirmed that p53 deficiency promotes tumorigenic transformation *in vitro*. Inhibiting SIR can prevent tumorigenesis in p53-deficient tissues. Meanwhile, SASP factors lead to tumor proliferation, migration, invasion, and metastasis, enhancing the malignant potential of tumor cells [20]. In addition, senescence-related factors regulate the tumor microenvironment and inhibit the anti-tumor effects of immune cells by promoting tumor angiogenesis. Inflammatory factors such as IL-6 and IL-8 promote immune escape by affecting NK cell and CD8^+^ T cell function. Furthermore, some senescent cells re-enter the growth cycle and acquire invasiveness through epigenetic modifications or p53 deficiency, a phenomenon known as senescence escape [14].

## SMP30 and tumors

### Structure and function of SMP30

The gene of SMP30 is located in the pl1.13-ql1.12 region of the X chromosome, consisting of seven exons and six introns, with a total length of approximately 17.5 kb. The single open reading framework of its cDNA contains 897 nucleotides, encoding 299 amino acids [21]. Structurally, SMP30 contains conserved Glu/Asp rich domains and calcium binding sites, which enable it to play important roles in various biological functions within cells. The calcium binding properties of SMP30 enable it to play a critical role in cellular signaling, metabolic regulation, and antioxidant responses. These functions contribute to the occurrence and progression of cellular senescence and related diseases [22]. In terms of subcellular localization, SMP30 mainly locates in the cytoplasm, but is also found in the nucleus and mitochondria. This distribution suggests that SMP30 functions in various cellular regions: regulating metabolism in the cytoplasm, influencing gene expression in the nucleus, and contributing to energy metabolism and oxidative stress response in mitochondria. Studies have shown that SMP30 exerts a protective effect against cellular senescence and related pathological processes, alleviating cell damage by regulating intracellular Ca^2+^ concentration and antioxidant signaling pathways [23]. The expression of SMP30 is significantly reduced in retinal ganglion cells stimulated by high glucose, while its overexpression can inhibit apoptosis and oxidative stress [24]. In addition, SMP30 is expressed in different tissues and cells, especially in important organs, such as the liver and kidneys, where it shows high expression levels. This is closely related to its role in regulating metabolism and maintaining cell function in these tissues. As age increasing, the expression of SMP30 gradually decreases, which is closely related to various pathological states, such as non-alcoholic fatty liver disease (NAFLD) and chronic obstructive pulmonary disease (COPD). The occurrence of these diseases is closely related to cellular senescence, inflammatory response, and metabolic disorders [25]. SMP30 is an important cellular marker, whose unique molecular structure and broad subcellular localization enable it to perform multiple functions in cellular physiology and pathology. This provides new insights into the mechanisms of senescence and treatment of related diseases.

### Expression and tumor prognosis prediction of SMP30 in tumors

**Figure 1:**
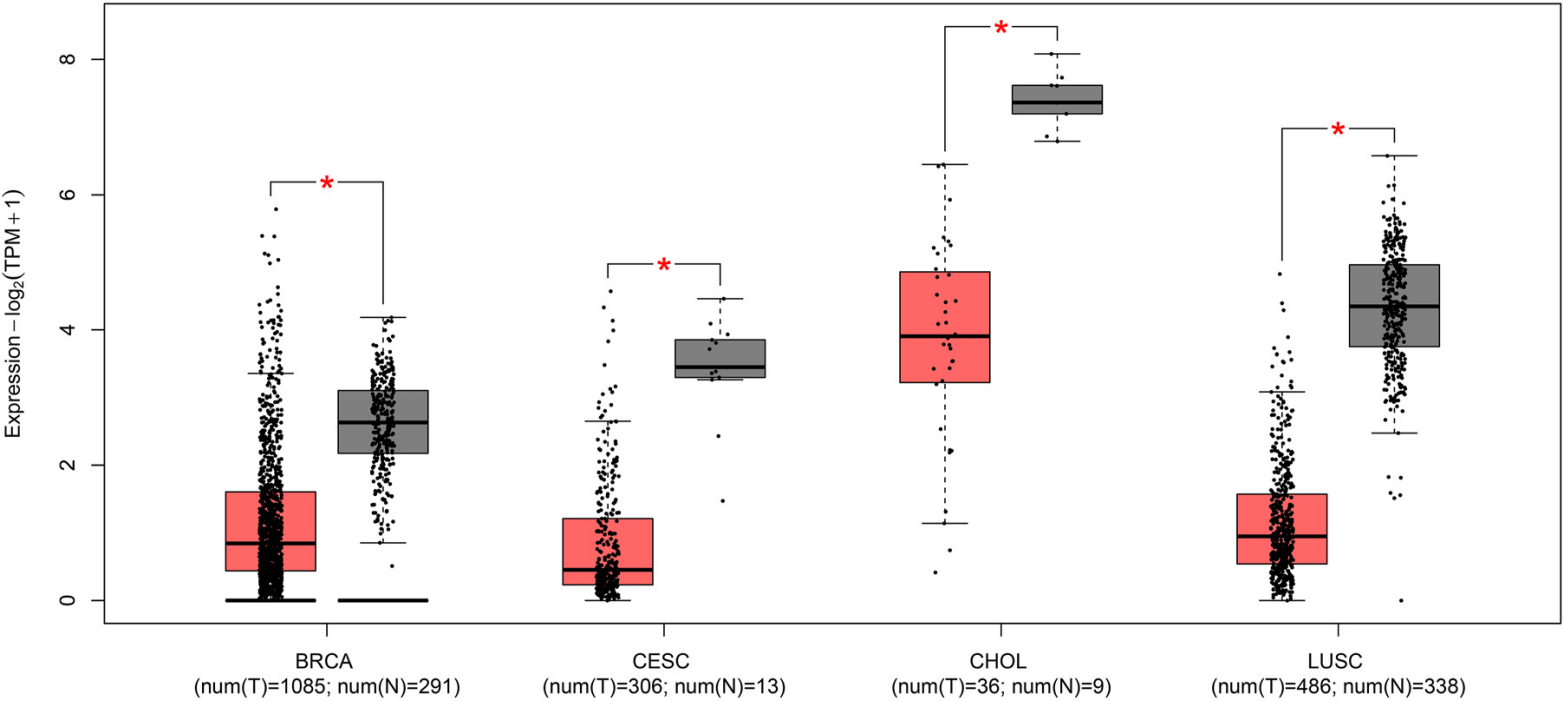
Expression of SMP30 in four tumors. According to TCGA database, expression of SMP30 significantly reduced in bladder urothelial carcinoma, breast invasive carcinoma, cervical squamous cell carcinoma and endocervical adenocarcinoma, cholangiocarcinoma, colon adenocarcinoma, kidney renal clear cell carcinoma and lung squamous cell carcinoma and other 16 kinds of tumors, (p<0.05). Four tumors of them were provided. BRCA (Breast invasive carcinoma, n of tumor group is 1,085, and control is 291). CESC (Cervical squamous cell carcinoma and endocervical adenocarcinoma, n of tumor group is 306, and control is 13). CHOL (Cholangio carcinoma, n of tumor group is 36, and control is 9). LUSC (Lung squamous cell carcinoma, n of tumor group is 486, and control is 338). *p<0.05 indicates statistical significance.

SMP30 is one of the hallmark molecules of cellular senescence, and its expression exhibits significant differences in different tumors. In TCGA database (http://gepia2.cancer-pku.cn/#index), expression of SMP30 significantly reduced in about 20 kinds of tumors (p<0.05), four of which were provided in Figure 1. Independent studies have also yielded consistent results. In hepatocellular carcinoma (HCC), It has been confirmed that lower expression of SMP30 was observed in tumor tissues compared to adjacent normal tissues. Decreased SMP30 is significantly associated with larger tumor size (p=0.012), advanced TNM stage (p=0.009), and shorter survival time (p<0.0001). Cox regression analysis found that decreased SMP30 is an independent risk factor for reduced overall survival in HCC patients (p=0.001). This indicates that SMP30 serves as an important negative prognostic indicator for HCC [26]. In non-small cell lung cancer (NSCLC), researchers performed immune-histochemical analysis on tumor tissues and adjacent normal tissues from 341 patients and found that SMP30 expression was significantly downregulated in NSCLC tissues. As in HCC, low expression of SMP30 is associated with poor overall survival time in NSCLC patients. a study on 341 patients showed that patients with low SMP30 expression had significantly lower overall survival (OS), with a median OS of 18 months, compared to 67 months in high expression group [27]. Furthermore, a study inducing liver tumors in adult zebrafish showed reduced SMP30 expression in lesions, hepatocellular carcinoma, cholangiocarcinoma, and mixed tumors compared to adjacent tissues, suggesting a general correlation between SMP30 expression and tumor occurrence and prognosis [28]. However, in breast cancer, Baek SM [29] found that SMP30 was specifically overexpressed in tumor glandular epithelial cells, and positively correlated with the malignancy and proliferation of glandular epithelial cells. It was lower in normal breast tissue and well-differentiated adenoma tissue. This differs from the expression of SMP30 in HCC and non-small cell lung cancer (NSCLC), as well as in invasive breast cancer of TCGA database. This difference may result from different tumor patterns, as well as differences in study subjects (race, age, etc.) or research methods. SMP30 is expressed at low levels in most tumor tissues, its expression in breast cancer requires further experimental confirmation.

### Regulation of SMP30 expression

**Figure 2:**
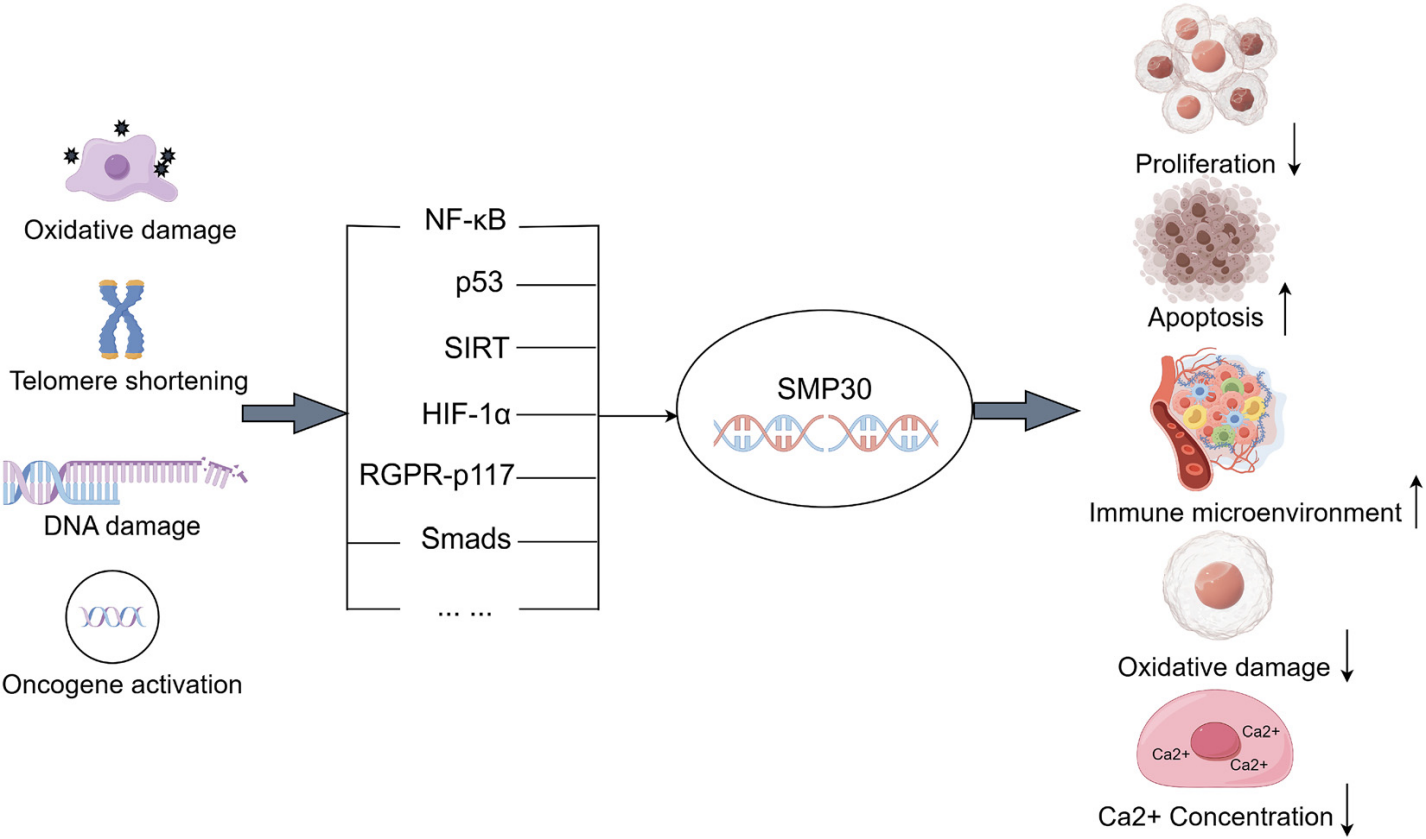
Expression of SMP30 was regulating by multiple signaling factor. Oxidative damage, telomere shortening, DNA damage and oncogene activation regulates expression of SMP30 through multiple signal pathways, including NF-κB, p53, HIF-1α, RGPR-p117 and so on. The increased expression of SMP30 can inhibit tumor cells proliferation, promote cancer cell apoptosis, regulate the immune microenvironment, and reduce intracellular Ca^2+^ concentration and oxidative stress.

SMP30 is regulated by various biological factors. Notably, it shows significant changes during the senescent process. SMP30 is significantly higher in young individuals than in elderly, and decreases gradually with age. This age-related decline of SMP30 contributes to a decline in cellular function and onset of age-related diseases [25]. The expression of SMP30 is regulated by various physiological conditions and transcription factors, such as Ca^2+^ concentration, NF-κB, p53, HIF-1α, SIRT, and so on [30], 31]. p53 plays a crucial role in cell cycle regulation and responses to DNA damage. When cells are subjected to stress or damage, activation of p53 can induce them to enter a senescent state, affecting expression of SMP30 [32]. NF-κB is a key signaling factor involved in the regulation of inflammatory responses and cell survival. Studies have shown that NF-κB affects the cellular senescence of cells by regulating the expression of SMP30 [33]. Furthermore, epigenetic modifications play an important role in the regulation of SMP30 expression. Research has shown that epigenetic changes, such as DNA methylation and histone modifications, can significantly affect the transcriptional activity of the SMP30 gene. Similarly, antioxidants and certain phytochemicals, such as Epigallocatechin Gallate (EGCG), regulate SMP30 expression by influencing epigenetic modifications and reducing oxidative stress-induced damage [34], 35]. The regulation of SMP30 is complex and diverse, involving the age-related decline, regulation by transcription factors, and influence of epigenetic modifications. Sequence analysis of the SMP30 promoter region revealed the presence of putative HRE binding motifs. Further analysis of the SMP30 promoter region with 5′ deletion and point mutants of HRE binding motifs showed that the HRE binding site was critical for high promoter activity. In addition, HIF-1α protein binds directly to the HRE binding motifs within the SMP30 promoter *in vivo*, and regulates SMP30 gene expression. These findings suggest that SMP30 is the novel target gene of HIF-1α, and SMP30 gene expression in hypoxia can be regulated by the control of HIF-1α expression [36]. The interaction of these mechanisms provides a new perspective on the role of SMP30 in cellular senescence and offers potential targets for the treatment of age-related diseases. Elucidating the regulatory mechanism of SMP30 on tumor cells proliferation, invasion, metastasis, and metabolic reprogramming will provide strong support for targeted tumor therapy. The factors which contribute to cell regulation via SMP30 were described in Figure 2.

## Mechanism of SMP30 in regulating tumor cells proliferation

### SMP30 and cell cycle

Calcium homeostasis imbalance plays an important role in tumor cells proliferation, especially in the development of chemotherapy resistance [37]. In epithelial ovarian cancer cell lines, the intracellular Ca^2+^ concentration of cisplatin resistant cells (MDAH-2774/DDP) is significantly reduced compared to parental cells. Ca^2+^ signaling is controlled in a cell cycle-dependent manner. SMP30 plays an important role in tumor cell cycle regulation through regulating intracellular calcium homeostasis [38]. In human HepG2 liver cancer cells, extracellular SMP30 was found to have suppressive effects on cell proliferation *in vitro* [39]. By modulating cyclin D1/CDK4/6-Rb pathway, interacting with CDK inhibitors, and controlling DNA damage checkpoints, SMP30 inhibits tumor cells proliferation. Studies have shown that SMP30 affects epithelial-mesenchymal transition (EMT) by interacting with Rho-associated protein kinase 1 (ROCK1). SMP30 influences the transition of tumor cells from the G1 to S phase by regulating the cyclin D1/CDK4/6-Rb signaling pathway, where cyclin D1 binds CDK4/6 to phosphorylate Rb, release E2F transcription factor, and initiate S phase gene expression and DNA synthesis. During cell senescence, SMP30 expression significantly decreases, which reduces the activity of cyclin D1 and CDK4/6, slowing cell cycle progression. [34]. In addition, SMP30 interacts with CDK inhibitors p21 and p27. p21 and p27 are key cell cycle regulatory factors that directly inhibit CDK activity, preventing progression of the cell cycle. Research has found that SMP30 indirectly affects cell proliferation by regulating the expression of p21 and p27. Overexpression of SMP30 inhibits p21 expression and promotes cell cycle progression, whereas its downregulation upregulates p21 and inhibits cell proliferation [25]. DNA damage can activate DNA damage checkpoint responses, leading to cell cycle arrest and allowing cells to repair the damage. SMP30 may promote the antioxidant capacity of cells and alleviate cell cycle arrest caused by DNA damage by enhancing the activity of the Nrf2/ARE (Antioxidant Response Element) signaling pathway [28]. Therefore, SMP30 not only participates in cell cycle regulation but also plays an important role in maintaining intracellular environmental stability and protecting cells from external damage.

### SMP30 and apoptosis

A protective effect of SMP30 against apoptosis has been observed [40], and this phenomenon has been partially attributed to the ability of SMP30 in regulating cellular Ca^2+^ concentrations, maintaining cellular homeostasis and preventing the cascade of events that lead to programmed cell death. Persistently elevated intracellular Ca^2+^ levels can induce cell apoptosis [8]. Zhou M [41] induced mitochondrial dysfunction and death in cancer cells by designing nanoparticles to create Ca^2+^ overload in tumor cells. This confirms that regulating (especially increasing) intracellular Ca^2+^ concentration can serve as a potential pro-apoptotic therapeutic strategy. SMP30 is involved not only in the mitochondrial apoptosis pathway and caspase activation pathway, but also in death receptor-mediated apoptosis signaling. Clarifying the mechanism of SMP30 in tumor cells apoptosis and utilizing its regulatory effects to promote tumor cells apoptosis is a potential therapeutic strategy for improving the prognosis of cancer patients. SMP30 can prevent calcium overload induced apoptosis by maintaining Ca^2+^ balance. SMP30 can enhance the activity of plasma membrane calcium pumps and regulate intracellular calcium concentration [42]. Research has demonstrated that SMP30 affects mitochondrial apoptosis by regulating the expression of Bcl-2 family proteins, which are important regulators of cell apoptosis. The balance between Bcl-2 and Bax directly affects cell survival and cell death. In the senescent state of cells, overexpression of SMP30 leads to increased expression of Bax, promotes the permeability of the mitochondrial outer membrane, triggers the release of pro-apoptotic factors in cells, and induces apoptosis [43]. Additionally, SMP30 interacts with the caspase pathway to further regulate the process of cell apoptosis. Caspases are a family of cysteine proteases that play a critical role in cell apoptosis signaling. It has been shown that SMP30 can affect the activity of caspase-3 and caspase-9, regulating the execution stage of cell apoptosis. SMP30 not only participates in cell survival signaling but also promotes cell apoptosis when cells are subjected to stress or damage [44]. SMP30 also plays an important role in the death receptor-mediated apoptosis pathway. Death receptors such as Fas and TNF receptors activate downstream signals through the binding of their ligands, inducing cell apoptosis. SMP30 modulates the signal transduction of death receptors by regulating the activity of related adapter proteins and downstream caspases, enhancing or inhibiting this apoptotic signaling pathway depending on the cellular context. Overexpression of SMP30 can enhance apoptosis signaling mediated by Fas and TNF receptors, leading to apoptosis of tumor cells [45]. SMP30 exhibits a context-dependent, dual role in regulating tumor cell apoptosis. It can function as either a pro-survival factor, protecting cells from death, or a pro-apoptotic trigger, depending on specific cues from the cellular environment, such as the tumor type, microenvironmental signals and the cell’s stress status.

## Effect of SMP30 on tumor cells metabolism

### SMP30 and glycol-metabolism

Glycolipid metabolism of tumor cells is the core driving force behind their malignant growth, manifesting as profound “metabolic reprogramming”. Tumor cells prefer to rapidly decompose glucose and produce large amounts of lactic acid through aerobic glycolysis, even in the presence of sufficient oxygen [46]. SMP30 regulates the expression of two key glycolytic enzymes, hexokinase 2 (HK2) and pyruvate kinase M2 (PKM2) in cellular glucose metabolism. This regulation is closely related to the Warburg effect, a phenomenon in which tumor cells preferentially utilize glycolysis over oxidative phosphorylation under aerobic conditions, resulting in increased lactate production [24]. SMP30 modulates the Warburg effect by influencing the AMPK/mTOR signaling pathway. AMP-activated protein kinase (AMPK) is a key regulator of cellular energy sensing, while mTOR is involved in controlling cell growth and metabolism. Research has found that SMP30 is positively correlated with AMPK activation. Activated AMPK inhibits the mTOR signaling pathway, which leads to reduced cellular glucose metabolism and tumor cells proliferation [14]. Under conditions of oxidative stress or nutrient deprivation, expression of SMP30 significantly increases. This upregulation enhances AMPK activity, inhibits mTOR pathway activation, and decreases the expression of HK2 and PKM2. This regulatory mechanism plays an important role in tumor development, because tumor cells often depend on enhanced glucose metabolism to support their growth and metastasis. Furthermore, SMP30 influences glucose metabolism by modulating oxidative stress-related signaling pathways. In high glucose environment, SMP30 expression decreases, which reduces cellular resistance to oxidative stress and promotes apoptosis and inflammatory responses. These findings suggest that SMP30 may affect the tumor microenvironment and growth by regulating both the antioxidant capacity and glucose metabolism of tumor cells [28].

### SMP30 and lipid metabolism

Abnormally active *de novo* lipogenesis in tumor cells is a crucial metabolic characteristic for maintaining their rapid proliferation and bio-membrane synthesis, SMP30 playing a key role in regulating this process. SMP30 is negatively correlated with the expression of key enzymes involved in adipo-genesis. In zebrafish embryo models, exposure to environmental pollutants such as perfluorooctanoic acid (PFOA) induces hepatic steatosis and upregulates lipid synthesis-related genes, a process accompanied by altered SMP30 gene expression. PFOA exposure upregulates the expression of genes such as fatty acid synthase (FASN) and acetyl-CoA carboxylase (ACC), which are core executors of *de novo* lipogenesis. SMP30 suppresses the expression of lipo-genic enzymes FASN and ACC by regulating the sterol regulatory element-binding protein (SREBP) pathway [47]. It has shown that SMP30-deficient mice exhibit impaired activation of FASN and steroid regulatory element binding protein-1c (SREBP-1c) under a high fat diet. This impairment is closely associated with excessive cholesterol accumulation in the liver. Long term vitamin C deficiency can inhibit lipogenesis, a process also regulated by SMP30. As a protein that regulates vitamin C biosynthesis, SMP30 plays a crucial role in lipid metabolism, and its deficiency typically causes disorders of lipid metabolism. Furthermore, SMP30 deficiency can affect the expression of enzymes involved in cholesterol synthesis, leading to abnormal cholesterol metabolism and an increased risk of hepatic steatosis, which is closely related to the development of non-alcoholic fatty liver disease (NAFLD) [48]. Meanwhile, SMP30 also involves in regulating the recycling and degradation of the cell surface low density lipoprotein receptor (LDLR), influencing the uptake of exogenous cholesterol. A study on obesity-related subclinical atherosclerosis found that serum SMP30 levels were positively correlated with vitamin D levels, while the obese group exhibited higher serum lipid profiles (including cholesterol). It established an association between SMP30 and systemic cholesterol metabolic status [49]. In tumor cells, SMP30 modulates cholesterol content, leading to alterations in membrane fluidity, which affects the lateral diffusion, dimerization, and activation of various transmembrane receptors, such as the epidermal growth factor receptor (EGFR). Cholesterol is a major component of lipid rafts, where many important signaling molecules tend to enrich, including certain proteins in the PI3K/AKT pathway. Therefore, SMP30’s regulation of cholesterol homeostasis may alter the assembly and stability of lipid rafts, impacting the activity of key pro-survival and proliferative signaling pathways such as EGFR and PI3K/AKT [47]. These pathways extensively intersect with cellular metabolic regulatory networks. Activated AKT can promote glycolysis and lipogenesis. By regulating cholesterol metabolism and membrane fluidity, SMP30 may serve as a hub connecting lipid metabolic reprogramming with the broad signaling transduction network in tumor cells, influencing tumor cells growth, invasion, and drug resistance.

## Impact of SMP30 on carcinoma microenvironment

### SMP30 and polarization of tumor-associated macrophages

It has been suggested that SMP30 plays an important role in regulating the balance of M1 and M2 macrophages, affecting the polarization of tumor-associated macrophages (TAMs). M1 macrophages are often associated with anti-tumor responses, while M2 macrophages are associated with tumor promotion and immune suppression. SMP30 may affect tumor development and progression by regulating the tumor microenvironment. Under the influence of SMP30, M1 macrophages are likely to inhibit tumor cells proliferation and metastasis by secreting various inflammatory factors. Upregulation of SMP30 has the potential to promote the activation of M1 macrophages and enhance their secretion of pro-inflammatory factors such as tumor necrosis factor alpha (TNF-α) and IL-6 [32]. On the contrary, downregulation of SMP30 tends to result in an increase in M2 macrophages, which in turn release anti-inflammatory factors and promote tumor growth and metastasis. Therefore, SMP30 may play a crucial role in the regulation of the balance of M1 and M2 macrophages. In addition, SMP30 can also affect macrophage polarization through NF-κB and STAT3 signaling pathways. The NF-κB signaling pathway is important for polarization of M1 macrophages, while the STAT3 signaling pathway is often associated with polarization of M2 macrophages. Research has found that SMP30 is positively correlated with the activity of NF-κB, which further suggests its potential role in regulating macrophage polarization [50]. SMP30 not only has the potential to affect polarization state of macrophages but also shapes the tumor microenvironment by regulating the cytokine profiles they secrete.

### SMP30 and immune checkpoint and costimulatory molecules

Metabolic reprogramming significantly influences tumor-immune interactions. Enhanced glycolysis, dysregulated lipid synthesis and oxidation, and altered amino acid metabolism collectively contribute to immune evasion by impairing T-cell activation, promoting regulatory immune cell populations, and modulating cytokine and checkpoint molecule expression [51]. SMP30 not only has the capacity to tumor cells metabolic reprogramming, but also potentially plays a significant role in shaping the tumor immune microenvironment by modulating the expression of immune checkpoint molecules and costimulatory molecules. Studies have revealed a strong correlation between SMP30 and various immune checkpoint pathways, which implies a potential regulatory relationship between them. In lung squamous cell carcinoma (LUSC), for instance, a high SMP30 expression group is associated with elevated Tumor-Infiltrating Dendritic Cells (TIDE) scores and dysfunction scores, alongside lower microsatellite instability (MSI) scores. This indicates that SMP30 might influence the functionality of immune checkpoint pathways such as PD-1/PD-L1 [52]. Mechanistically, SMP30 might affect PD-L1 expression by regulating calcium signaling or transcription factors NFAT. Overexpression of RGPR-p117 (a transcription factor which regulates SMP30) in breast cancer MDA-MB-231 cells downregulates pro-growth signaling molecules (e.g., Ras, PI3K, Akt, mTOR) while upregulating tumor suppressor proteins (p53, Rb, p21) [53]. Such alterations in key signaling pathways could indirectly impact PD-L1 expression levels. Furthermore, SMP30 is suspected to costimulatory molecules. In a co-culture system of 3T3-L1 adipocytes and RAW264.7 macrophages, Overexpression of SMP30 blocks inflammatory crosstalk and reduces the production of pro-inflammatory factors [54]. This anti-inflammatory effect is likely accompanied by an upregulation of costimulatory molecules, such as CD80 and CD86, which has the potential to foster a microenvironment more conducive to T cell activation. Notably, treatment of macrophages with the marine compound DHMBA (3,5-dihydroxy-4-methoxybenzyl alcohol) inhibits NF-κB signaling and inflammatory factor production while upregulating SMP30 expression [55]. This identifies SMP30 as a putative key downstream effector molecule within regulatory networks governing immune checkpoint molecule expression.

### SMP30 and angiogenesis

VEGF is a key factor in promoting tumor angiogenesis, which stimulates endothelial cell proliferation and migration by binding to VEGFR. SMP30, has been shown to be closely related to the upregulation of VEGF. Activation of SMP30 may enhance VEGF expression and promotes angiogenesis [45]. This regulation not only affects the vascular structure of the tumor microenvironment but also potentially alters the survival environment of tumor cells by providing essential nutrients and oxygen, promoting tumor growth and metastasis. The migration of endothelial cells and the formation of vascular lumens are two important steps in angiogenesis. SMP30 could enhance the growth and migratory abilities of endothelial cells, potentially promotes their localization and lumen formation in the tumor microenvironment, facilitates the formation of new blood vessels, and provides the necessary blood supply for tumor growth [56]. HIF-1α is a transcription factor crucial for the hypoxia response, which is activated under hypoxic conditions in the tumor microenvironment, promoting the expression of VEGF. SMP30 can regulate the activity of HIF-1α, affecting the expression of VEGF and its downstream signaling pathways. This mechanism provides a putative molecular basis for understanding how SMP30 could promote angiogenesis by regulating the hypoxia response in the tumor microenvironment [57].

## SMP30 and tumors metastasis

### SMP30 and EMT

EMT plays a crucial role in tumor metastasis [58]. Decreased SMP30 stimulates EMT and causes severe renal interstitial fibrosis at the end stage of CKD in Siberian tigers. SMP30 can be present as a new indicator of EMT signaling [59]. SMP30 regulates the EMT process through several mechanisms. These include affecting the expression of EMT markers, such as E-cadherin and N-cadherin, modulating EMT transcription factor activity, and interacting with the TGF-β/Smad signaling pathway. E-cadherin is a marker of epithelial cells, while N-cadherin marks stromal cells. In HCC cells, increased SMP30 expression leads to higher E-cadherin and lower N-cadherin levels. This suggests that SMP30 inhibits EMT progression and reduces the metastatic ability of tumor cells by regulating these markers [34]. Twist and Snail are EMT-promoting transcription factors, and their overexpression often correlates with increased tumor cells invasiveness [60]. Overexpression of SMP30 can downregulate the expression of Twist and Snail, inhibiting EMT progression and weakening the invasiveness of tumor cells [61]. TGF-β is one of the core regulatory cytokines in tumor cells EMT. In cells overexpressing SMP30, TGF-β-induced Smad activation is inhibited, further supporting the important role of SMP30 in EMT [62].

### SMP30 and extracellular matrix remodeling

Matrix metalloproteinases (MMPs) are important enzymes involved in extracellular matrix (ECM) degradation. MMP-2 and MMP-9 are widely recognized as playing critical roles in the tumor microenvironment [63]. SMP30 affects ECM remodeling by regulating the expression of MMP-2 and MMP-9. This regulatory effect may be closely related to the role of SMP30 in cellular senescence, a process often accompanied by changes in ECM components and abnormal expression of MMPs [45]. Fibronectin, a key extracellular matrix component, plays crucial roles in cell adhesion, migration, and proliferation. SMP30 is positively correlated with the synthesis of fibronectin, enhancing cell adhesion and migration abilities. Additionally, changes in laminin can affect the interaction between cells and the ECM, regulating cell behavior and the tumor microenvironment [64]. Integrins are receptors on the cell membrane which are crucial for signal transmission between cells and extracellular matrix. Moreover, abnormal activation of integrin signaling pathways is closely linked to the occurrence and progression of various tumors, particularly regarding tumor cells invasiveness and metastasis. Changes in SMP30 activate or inhibit the integrin signaling pathway, affecting cell growth, migration, and differentiation [65]. SMP30 plays an important role in the invasion and migration of tumor cells by regulating the activity of MMP-2 and MMP-9, and influencing the expression of fibronectin and laminin. Furthermore, SMP30 modulates integrin signaling pathways, which in turn affect ECM remodeling.

## Clinical applications and prospects of SMP30

Low expression of SMP30 is an important molecular marker of cell senescence and early tumor development. Therefore, SMP30 has potential clinical application value as an early diagnostic biomarker for tumor prognosis evaluation. The combined detection of anti-SMP30 antibody and alpha-fetoprotein (AFP) enhances diagnostic performance for HCC, with a positive rate of 69.23 % compared to 25–65 % sensitivity of AFP testing alone [66]. The clinical translation of SMP30-targeted strategies in tumor therapy shows promising potential, supported by advances in gene therapy, protein activation, and biomarker development. Gene therapy approaches utilizing dendritic cells (DCs) transduced with SMP30 expressing lentiviral vectors have demonstrated enhanced antitumor immunity against HCC *in vitro* and *in vivo*. These modified DCs exhibit increased expression of costimulatory molecules (CD80, CD86) and secrete elevated levels of cytokines such as IL-2, IL-12, and IFN-γ, which potentiate CD8+ T cell proliferation and cytotoxicity, resulting in significant tumor growth inhibition [67], 68]. This immunotherapeutic strategy highlights SMP30’s role as a tumor associated antigen with high specificity and immunogenicity. Additionally, the development of protein activation agents that upregulate SMP30 expression has shown efficacy in reducing tumor volume and promoting apoptosis, as observed with laminarin treatment in HCC models [69]. SMP30 also holds promise as a predictive biomarker for therapeutic response and prognosis. For example, decreased SMP30 expression correlates with poorer overall survival time in NSCLC patients, suggesting its utility in guiding individualized treatment plans [27]. Furthermore, innovative biosensor platforms capable of highly sensitive detection of SMP30 autoantibodies in serum facilitate early cancer diagnosis and monitoring, enhancing clinical management [70]. Combining SMP30-targeted therapies with existing immunotherapies and molecular targeted agents may synergistically improve treatment efficacy by enhancing immune activation and overcoming resistance mechanisms. Collectively, these advances underscore SMP30’s potential as a multifaceted therapeutic target and biomarker in cancer treatment, warranting further clinical investigation to optimize its application.

## Conclusions

SMP30 is a key factor in cellular senescence and plays an important role in tumor processes. It affects multiple mechanisms of tumors, such as the cell cycle, apoptosis, metabolic reprogramming, EMT, and immune escapi. This discovery offers a new perspective on tumor biology and opens new possibilities for clinical applications. It also provides potential benefits for tumor prevention and treatment. Firstly, SMP30 can inhibit abnormal proliferation of tumor cells and induce apoptosis by regulating intracellular Ca^2+^ concentration and affecting multiple signaling pathways, such as p53 and NF-κB. Secondly, SMP30 has antioxidant function, which can reduce the damage of reactive oxygen species (ROS) to DNA and lower the risk of mutation, indirectly playing a role in tumor prevention. Thirdly, SMP30 enhances the sensitivity of certain chemotherapy drugs, such as cisplatin, by regulating calcium-dependent enzymes, such as ATPase, improving treatment efficacy. Fourthly, the expression of SMP30 correlates with tumor staging or prognosis, and it has significant potential as a biomarker for adjunctive diagnosis or monitoring treatment efficacy. However, the clinical application of SMP30 still faces numerous issues and challenges. Firstly, the mechanism of action is not fully understood. The specific mechanisms of SMP30 still require further research. It is still unclear how it precisely regulates calcium signaling or interacts with other proteins. This lack of knowledge limits the development of targeted therapeutic applications. Secondly, the evidence mainly comes from cell experiments or animal models, lacking large scale clinical data support, and there remains a gap before clinical application. Thirdly, the effect of SMP30 may be tissue specific. In some tumors, it may even promote progression, necessitating careful evaluation of its double-edged sword nature. Fourthly, accurate detection of SMP30 in tissue or blood, as well as the development of safe drugs targeting its pathway, such as activators or inhibitors, still face technical challenges. Fifth, SMP30-targeting strategies hold great potential for overcoming drug resistance and improving treatment sensitivity. Modulating SMP30 activity may disrupt cancer cell survival mechanisms and restore responsiveness to anticancer drugs. However, translating these findings into clinical practice requires rigorous validation through well-designed preclinical studies and clinical trials to ensure safety, efficacy, and optimal therapeutic windows. Future research should focus on revealing the molecular regulatory network of SMP30 and specific target sites, while conducting large-sample population studies to evaluate its diagnostic, prognostic and therapeutic value in tumors.
